# Prognosis of Water Quality Sensors Using Advanced Data Analytics: Application to the Barcelona Drinking Water Network

**DOI:** 10.3390/s20051342

**Published:** 2020-02-29

**Authors:** Diego Garcia, Vicenç Puig, Joseba Quevedo

**Affiliations:** 1 Supervision, Safety and Automatic Control Research Center (CS2AC), Universitat Politécnica de Catalunya (UPC), Terrassa Campus, Gaia Research Bldg., Rambla Sant Nebridi, 22, Terrassa, 08222 Barcelona, Spain; kobydiego@gmail.com (D.G.); joseba.quevedo@upc.edu (J.Q.); 2 Aigues de Barcelona, Empresa Metropolitana de Gestió del Cicle Integral de l’Aigua S.A., 08028 Barcelona, Spain; 3 Institut de Robòtica i Informàtica Industrial (CSIC-UPC), Carrer Llorens i Artigas 4-6, 08028 Barcelona, Spain

**Keywords:** water quality monitoring, sensor prognosis, water distribution network

## Abstract

Water Utilities (WU) are responsible for supplying water for residential, commercial and industrial use guaranteeing the sanitary and quality standards established by different regulations. To assure the satisfaction of such standards a set of quality sensors that monitor continuously the Water Distribution System (WDS) are used. Unfortunately, those sensors require continuous maintenance in order to guarantee their right and reliable operation. In order to program the maintenance of those sensors taking into account the health state of the sensor, a prognosis system should be deployed. Moreover, before proceeding with the prognosis of the sensors, the data provided with those sensors should be validated using data from other sensors and models. This paper provides an advanced data analytics framework that will allow us to diagnose water quality sensor faults and to detect water quality events. Moreover, a data-driven prognosis module will be able to assess the sensitivity degradation of the chlorine sensors estimating the remaining useful life (RUL), taking into account uncertainty quantification, that allows us to program the maintenance actions based on the state of health of sensors instead on a regular basis. The fault and event detection module is based on a methodology that combines time and spatial models obtained from historical data that are integrated with a discrete-event system and are able to distinguish between a quality event or a sensor fault. The prognosis module analyses the quality sensor time series forecasting the degradation and therefore providing a predictive maintenance plan avoiding unsafe situations in the WDS.

## 1. Introduction

The quality of the drinking water, supplied by the Water Utilities (WU) to the citizens, is regulated by different entities to ensure full protection of public health [1]. In order to accomplish these regulations, WU monitors the Water Distribution System (WDS) placing water quality sensors and analyzers at different strategic locations. Moreover, experts of the WU, take samples periodically (also under regulation) at specific points of the network to analyze on-site. There are different types of water quality sensors, sensors that are able to monitor a single water quality parameter or multiple parameters.

The most common parameters monitored are temperature, chlorine, conductivity and pH. Other parameters such as turbidity, or total organic carbon (TOC) are also measured commonly. Which parameters to measure and how often is determined by the water quality department of the WU [2].

There are several techniques to treat the water in WDS and keep it healthy for human consumption. One common disinfection technique is the chlorination of water. This process consists of injecting chlorine or derivatives in the water. Thus, chlorine is one of the most important parameters to monitor because is used for disinfection purposes. The operator injects continuously a certain concentration of chlorine in the drinking water, usually in the reservoirs, by means of an automatic controller regulated by set-point [3]. A low concentration of chlorine can result in incomplete disinfection with consequent danger for the citizens’ health. However, high concentrations of chlorine may produce odor and may also increase levels of trihalomethanes (THMs) in the drinking water. Consequently, having an accurate measure of chlorine is very important. However, it is difficult because of the injected chlorine is consumed [4]. This consumption is related to reactions in the bulk water and in the pipe wall generating a biofilm (a group of microorganisms adhered to the surface of the pipes).

A standard amperometric chlorine sensor has a membrane and electrolyte to control the reaction of the chemical reduction of hypochlorous acid at the cathode. This causes a change in the current between the anode and the cathode that is proportional to the chlorine concentration. These sensors require a periodic maintenance plan to clean the solids that slowly accumulate in the membrane and to replace the electrolyte. The manufacturer specifies a frequency period for each maintenance action required.

Another important factor to consider when measuring the chlorine is the pH dependency. The relative amount of hypochlorous acid or hypochlorite present depends on pH. Thus, to achieve more accurate chlorine measurements, the pH measurement is required.

Taking into account the complexities mentioned, this paper is focused on developing a methodology that forecasts chlorine sensor’s loss of sensitivity to keep the sensor producing reliable data. This methodology allows the WU to increase data reliability reducing downtime and to establish a predictive maintenance plan reducing corrective actions.

Quality sensors require a continuous calibration following the procedures established by the manufacturer to produce reliable measurements. Additionally, a preventive maintenance plan according to the manufacturer recommendations is required to guarantee data reliability.

However, even applying the recommended preventive planning, quality sensors are prone to suffer from several problems (see Table 1). Therefore, a corrective plan is still required to address these unexpected problems affecting the availability and reliability of the sensor.

On the other hand, there already exists quite a lot of research regarding methods to detect and avoid contaminant injection in the water distribution networks guaranteeing the safety of the drinking water network [5,6,7]. In [8], a comparison of a set of sensors (from different manufacturers) assessing distinct quality parameters is carried out. This study examins the sensitivity of the different sensors in the presence of several contaminants. In [9], the hydraulic data and water quality are considered to minimize false positives numbers in the detection of quality events. In [10], several change-point detection algorithms are used to analyze the autoregressive model residual. The sensor placement of quality sensors is also an important issue to have a good quality monitoring performance but keeping low operational costs [11]. In [12], artificial neural networks (ANNs) are used to model the multivariate water quality parameters and detect possible outliers. In [13], the authors explore and compare two models for contaminant event detection in WDS: support vector machines (SVM) and minimum volume ellipsoid (MVE). The outputs of these two models are processed by sequence analysis to classify the event as a normal operation or an actual quality contaminant event. In [14], incorporates hydraulic information to detect events applying spatial analysis to complement the local analysis (for each sensor) with existing mutual hydraulic influences. In [15], local and spatial data analysis is performed using the simulation of contaminant intrusions. The proposed spatial model detects trends in the network based on finding similar and exceptional behavior in sensors that are located upstream. In [16], spatial models considering the correlations between observations are implemented to validate water consumption data coming from water flow sensors.

Model-based approaches, such as [7], have the main drawback that the performance depends directly on the water network model’s accuracy. Moreover, due to the complex behavior of the water parameters, it is unfeasible to develop an accurate physical model to describe the water quality dynamics.

Hence, data-driven approaches are very interesting in this case and therefore widely used.

One important drawback of data-driven approaches is the assumption that data gathered from these sensors are accurate and precise, such as data coming from simulations. However, as we have pointed out, raw data from quality sensors could not be ready to be analyzed or to extract solid conclusions. Unreliable water quality information is a serious problem for the WU to guarantee the citizens safety. Thus, a data cleaning process must be performed first, as [13] points out.

Hence, the main motivation of this work is to provide a data analytics methodology for monitoring quality sensors and events applicable to drinking water networks.

The contributions of this work are twofold. On the one hand, this work provides a procedure to get a solid information basis, discarding unreliable data, to improve the decision making of the WU in water quality management. On the other hand, a prognosis module estimates the remaining useful life (RUL) of water quality sensors located in the WDS allowing the WU to apply predictive maintenance.

The proposed methodology has been satisfactorily tested on the Barcelona drinking water network.

The structure of the paper is the following: In Section 2, the considered case study to illustrate the proposed methodology is introduced. In Section 3, the diagnosis and prognosis methodologies are described. In Section 4, the results obtained from three real scenarios of the considered case study are presented and discussed. Finally, in Section 5, the conclusions are provided as well a future research paths.

## 2. Case Study

**Figure 1:**
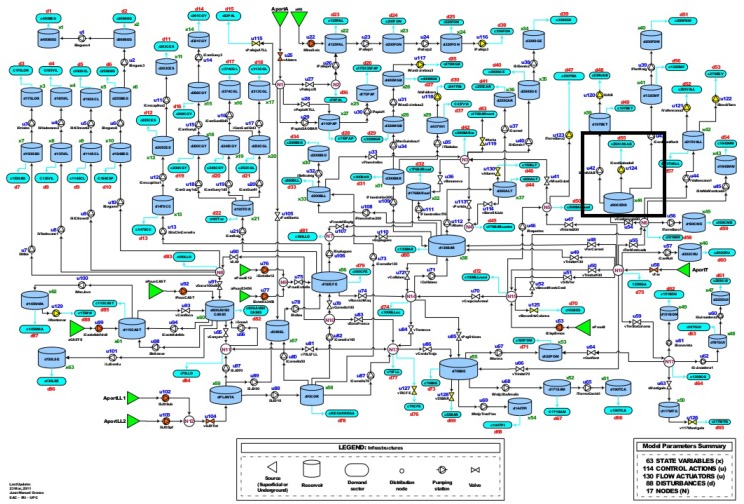
Barcelona Water Network.

To illustrate the proposed prognosis methodology a case study based on a part of the Barcelona water network is used. The Barcelona network is a complex water distribution system with more than 4600 km of pipes that supply drinking water to 218 sectors of demand (see Figure 1). In this network, there are 200 quality sensors and analyzers in charge to guarantee water quality. Moreover, a laboratory sample daily several points of the network to do more in-depth analyses.

**Figure 2:**
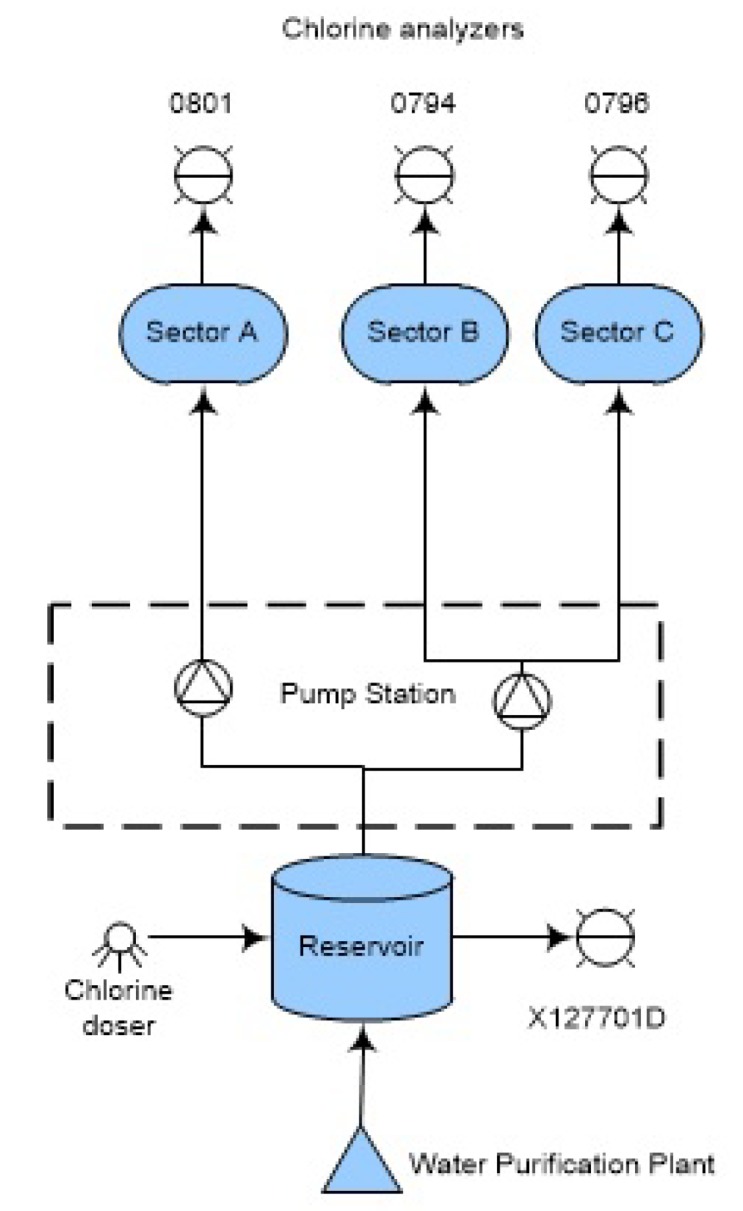
Case study from the Barcelona Water Network.

This paper is focused on the zone highlighted with a rectangle in Figure 1 and depicted in Figure 2 for illustrative purposes.

The water supplied in this zone can come from two different water purification plants that extract water from the rivers Ter and Llobregat. Since the mineral composition of these rivers is very different water quality can vary significantly depending on which plant the water comes. The water arriving from these plants is stored in a tank to be served to the three associated demand sectors when required. The chlorine injection is done in this tank with an automatic system to keep the concentration at the set-point established according to sanitary regulations. On the other hand, At each demand sector entrance, a multi-parametric quality analyzer is available to continuously monitor the water quality and in particular the chlorine concentration. These analyzer supply date every 15-min to the quality monitoring center. The parameters monitored by these analyzers are temperature, conductivity, pH and chlorine.

The water quality data collected by the sensors are analyzed by the experts using visualization software to check if there exists any quality event or problem. Then, the experts check the chlorine concentrations measured using the sensors with the samples analyzed in the laboratory.

The methodology presented in this paper has been based on the knowledge of the experts used to analyze. This methodology allows checking and even forecasting problems in the quality of the water network.

## 3. Methodology

A diagnosis module has been designed to detect and diagnose the sensor health status. This module is briefly detailed next, however, further details can be found in [17]. Moreover, a prognosis methodology has been developed to forecast the loss of sensitivity in chlorine sensors of the WDS.

### 3.1. Diagnosis

This module is in charge of detecting and classifying events affecting the water quality parameters by means of the analysis of local and spatial data. For each sensor, the analysis of local data is carried using an Artificial Neural Network (ANN) to model the behavior of the water quality time series. This model provides a prediction of the current value of the sensor based on past measurements provides as inputs to the ANN. This model is able to detect abrupt changes in the time series, but can not differentiate if this change is due to fault or a quality event. These two different situations can be distinguished by using several sensors that are spatially correlated. The predecessor (PD) spatial model checks the consistency between the sensor located downstream and the one located upstream. In the considered case study, the upstream sensor is the chlorine analyzer located in the tank where the chlorine is injected while the downstream ones are located at the entrance of the demand sectors.

Indeed, this is the procedure followed by the WU experts. First, they look for anomalous behaviors in the signals and next they validate their conclusions looking for information from other sensors hydraulically related to conclude if it is only a sensor problem or a real water quality problem.

Following a procedure similar to those used by the human experts that analyze the quality measurement, a fault diagnosis procedure is developed. This procedure works as follows: the consistency of each local and spatial model is checked by generating a residual that is checked against a threshold. The consistency check generates a 0 if the residual is below the threshold and 1 otherwise. This threshold is created by defining a lower bound $\tau^{\mathrm{LB}}$ and upper bound $\tau^{\mathrm{UB}}$ according to [18] as follows:

(1) $$\begin{array}{c} \begin{array}{cc} \tau^{\mathrm{LB}} & =Q_{1}-3\;\cdot \;IQR\\ \tau^{\mathrm{UB}} & =Q_{3}+3\;\cdot \;IQR \end{array} \end{array}$$

where $Q_{1}$ and $Q_{3}$ are the first and third quartiles, respectively, and IQR is the interquartile range (the difference between the third and first quartiles) obtained from the residuals of the training data set.

Finally, the combination of the binarized residuals are the signature of the sensor’s state according to the Table 2.

**Figure 3:**
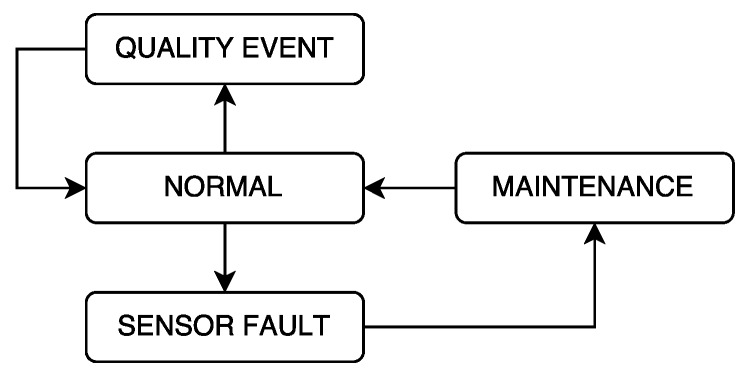
State diagram of a quality sensor.

The fault diagnosis algorithm described above can be represented as a state machine (discrete-event system). The state diagram is presented in Figure 3. Assuming that the sensor starts in the normal (non-faulty) state, two possible situations can occur; a sensor fault or a quality event. In case a sensor fault occurs, after it is detected, the sensor fault state is reached. Finally, if the sensor is deactivated enter the maintenance state. Finally, after the sensor is repaired, it returns to the normal state. On the other hand, in case a quality event occurs, it can be caused by an intended action (e.g., hydraulic action, chlorine reference change) or by some unexpected infiltration.

According to Table 2, a sensor is in non-faulty situation when all residuals are within their thresholds. On the other hand, a quality event can be identified when the ANN residual violates its threshold but not the PD one. Finally, when the PD residual violates its residual, a sensor fault is diagnosed independently of the ANN residual.

### 3.2. Prognosis

This module forecasts the Remaining Useful Life (RUL) based on a predetermined Failure Threshold (FT). As proposed in [19], the RUL is given by:

(2) $$\mathrm{RUL}\in N\;|\;\hat{y}\left(t+\mathrm{RUL}|t\right)=\mathrm{FT},$$

where $\hat{y}\left(t+\mathrm{RUL}|t\right)$ is the RUL-step ahead forecast at time *t* of a given predictive model $\hat{y}$.

A data-driven approach is used to derive the predictive models from the data collected. Three different methods have been considered for multi-step forecasting the chlorine decay: Brown’s double exponential smoothing, drift and Holt’s linear filter.

The main contribution of this module is to consider the uncertainty of the models’ estimations. In order to compute the uncertainty of each model, it is trained for a set of horizons obtaining the optimal parameters for each forecast horizon in order to improve the models’ forecast performance while decreasing the residuals’ variance generated by the models.

The multi-step forecasting approach consists in fitting a model with the form

(3) $$\hat{y}\left(t+h|t,\theta_{h}\right)$$

where $\theta_{h}$ is the vector of parameters to adjust for each forecast horizon in 1≤h≤H with a maximum forecast horizon *H*. Once a model is fitted for each horizon, a set of models are obtained for each method

(4) $$Y=[{\hat{y}}_{1},{\hat{y}}_{2},\ldots ,{\hat{y}}_{h},\ldots ,{\hat{y}}_{H}]$$

where ${\hat{y}}_{h}$ is given by Equation (3) using a simplified notation and $Y\in \{Y_{B},Y_{D},Y_{H},Y_{\mathrm{NNET}},Y_{\mathrm{QRF}},Y_{\mathrm{SVM}}\}$ meaning Brown, drift, holt, artificial neural networks, quantile random forests and support vector machines methods, respectively. These methods are detailed next.

### 3.3. Forecast Models

The Brown’s double exponential smoothing model can be expressed as follows:

(5) $$y_{1}\left(t\right)=\alpha_{h}y\left(t\right)+\left(1-\alpha_{h}\right)y_{1}\left(t-1\right)$$

(6) $$y_{2}\left(t\right)=\alpha_{h}y_{1}\left(t\right)+\left(1-\alpha_{h}\right)y_{2}\left(t-1\right)$$

(7) $$a=\alpha_{h}\frac{h}{1-\alpha_{h}}$$

(8) $${\hat{y}}_{h}\left(t+h|t\right)=\left(2+a\right)y_{1}\left(t\right)-\left(1+a\right)y_{2}\left(t\right),$$

where *h* is the forecast horizon and α is the smoothing parameter.

The unique parameter to be optimized for each horizon *h* is

(9) $$\theta_{h}=\left\{\alpha \right\}$$

The drift model provides a simple way to estimate the change over time from a set of observations. Indeed, it estimates the drift between the first observation and the *m* previous one as follows

(10) $${\hat{y}}_{h}\left(t+h|t\right)=y\left(t\right)+h\left(\frac{y\left(t\right)-y\left(t-m_{h}\right)}{m_{h}}\right),$$

where $m_{h}$ is the distance between the actual observation and the previous one for a given horizon *h*.

The set of parameters to be optimized for this model is

(11) $$\theta_{h}=\left\{m\right\}$$

The Holt’s linear method in the state-space form is the third considered modeling approach [20].

The state-space forecast general representation has the following form

(12a) $$\hat{y}\left(t+h|t\right)=wx\left(t\right)+\epsilon_{h}\left(t\right),$$

(12b) $$x\left(t\right)=Fx\left(t-1\right)+g_{h}\epsilon_{h}\left(t\right),$$

where x(t)=[l(t)b(t)] is the state vector composed by the level l(t) and the growth rate b(t), w=[1h], $F=\left[\begin{array}{cc} 1 & 1\\ 0 & 1 \end{array}\right]$, $g_{h}=\left[\alpha_{h}\;\beta_{h}\right]$ and $\epsilon_{h}\left(t\right)$ is a random error with zero mean.

The performance of the model, as showed in [20], depends directly on the initial state x(0). In this model, the set of parameters to be optimized for each horizon *h* are

(13) $$\theta_{h}=\left\{\alpha ,\beta ,l\left(0\right),b\left(0\right)\right\}.$$

Multilayer Perceptron (MLP) Networks is a type of feedforward artificial neural network consisting of an input layer, one or multiple hidden layers, and an output layer, i.e., the model prediction. This work considers only single-hidden-layer feed-forward neural networks (NN) with *H* hidden neurons. These kinds of networks are used to predict different continuous physical processes [21]. Each layer is composed of one or multiple neurons and the layers are connected one-by-one where each neuron has a direct connection to the neurons of the subsequent layer (i.e., without cycles). The basic idea of the NN construction is to adjust the corresponding weights for each link connection between neurons minimizing an error function of the prediction using a training dataset. A simplification of the mathematical background of the NN expression [22] to forecast the *h* ahead value at instant *t* is

(14) $${\hat{y}}_{h}\left(t+h|t\right)=f\left(x\left(t\right),w_{h}\right),$$

where x(t)=[y(t),y(t−1),…,y(t−N−1)] is the vector with the *N* previous values of the actual time series at time instant *t* and $w_{h}$ is the vector of the weights assigned to each neuron connection for a forecast horizon *h*.

Hence, the parameters to be adjusted in the NN models are

(15) $$\theta_{h}=\left\{H,w\right\}$$

Random Forests (RF) are a powerful and popular machine learning tool for high dimensional classification and regression [23]. RF are a combination of tree predictors that vote for the most popular class for classification or provide the average of the trees predictors for regression. Given an input *x*, a tree predictor T(x,Θ) provides a categorical value (classification) or a continuous value (regression). Basically, the prediction trees sub-divide the complex input space into smaller partitions, recursively, in order to obtain small cells where a simple model or even a constant value (the average) can represent the cell group. It starts at a root and the final cells are the leaves. How to split and which features are involved in each split is part of the training phase. The structure of the tree is represented by Θ.

Quantile Regression Forest (QRF) is a generalization of RF, as it provides not only the conditional mean, but also estimates the conditional quantiles [24]. The RF final prediction (in the regression form) for a given new input *x* is made averaging the predictions from all the *B* individual regression trees

(16) $${\hat{y}}_{h}\left(t+h|t\right)=\frac{1}{B_{h}}\sum_{b=1}^{B_{h}}T\left(x\left(t\right);\Theta_{bh}\right),$$

where is the individual tree regression function and $\Theta_{bh}$ characterizes the *b*-th random forest tree for a given forecast horizon *h*. The input vector x(t)=[y(t),y(t−1),…,y(t−N−1)] is the vector with the *N* previous values of the actual time series at time instant *t*, and $B_{h}$ is the number of random forest trees.

Hence, the QRF parameters to be tuned are

(17) $$\theta_{h}=\left\{B,\Theta_{b}\right\}$$

RF have been implemented using the R package *rpart* [25].

The goal of Support Vector Machines (SVM) is to find a function f(x) given a training dataset $\{\left(x_{1},y_{1}\right),\ldots ,\left(x_{m},y_{m}\right)\}\subset X\times R$ where X is the space of the input predictors and $y_{i}$ the target. In case of a linear function *f*, it takes the form

(18) f(x)=〈w,x〉+b,

where w∈X,b∈R and 〈·,·〉 denotes the scalar product in X. For nonlinear functions, the input space is mapped first into a new feature space F using a mapping function Φ:X→F [26]. The forecast expression of SVM is

(19) $$\hat{y}\left(t+h|t\right)=\sum_{i=1}^{L_{h}}\left(\alpha_{ih}-\alpha_{ih}^{*}\right)k\left(x_{i}\left(t\right),x\left(t\right)\right)+b_{h},$$

where $\alpha_{i}$ and $\alpha_{i}^{*}$ are Lagrange multipliers and $k(x_{i}\left(t\right),x\left(t\right))$ is the mapping function, known as the kernel function, x(t)=[y(t),y(t−1),…,y(t−N−1)] is the vector of the *N* previous values of the actual time series and $x_{i}\left(t\right)$ is the element *i* of the input vector, i.e., y(t−i+1).

Hence, the parameters to be adjusted in the SVM models are

(20) $$\theta_{h}=\left\{L,\alpha_{i},\alpha_{i}^{*},b\right\}$$

### 3.4. Models Performance Metric

Two different metrics are used to assess the model’s performance. On the one hand, for the linear models, the training stage finds the optimum parameter values for each model minimizing, as a function cost, the mean absolute percentage error (MAPE) defined as

(21) $$\min\;\frac{1}{n}\sum_{t=1}^{n}\left|\frac{y\left(t+h\right)-\hat{y}\left(t+h|t,\theta_{h}\right)}{y(t+h)}\right|,$$

where $\theta_{h}$ is the vector of parameters for all 1≤h≤H of each linear model to be optimized according to Equations (9), (11) and (13), respectively.

On the other hand, for the nonlinear models, the training stage finds the optimum parameter values for each model minimizing the root mean square (RMSE) defined as

(22) $$\min\;\sqrt{\frac{1}{n}\sum_{t=1}^{n}{\left[y\left(t+h\right)-\hat{y}\left(t+h|t,\theta_{h}\right)\right]}^{2}}$$

where $\theta_{h}$ is the vector of parameters for all 1≤h≤H of each nonlinear model to be optimized according to Equations (15), (17) and (20), respectively.

Moreover, the training of nonlinear models is performed with *k*-fold cross-validation to avoid the over-fitting of the models. *K*-cross-validation splits the dataset randomly into *k* equal subsamples. One of these *k* subsamples is used for validation and testing and the rest is used for training the model. The cross-validation is then repeated *k* times using each sample only once.

### 3.5. Prognosis Performance Evaluation

In order to evaluate the prognosis models performance, the Prognosis Horizon (PH) is computed as

(23) $$\mathrm{PH}=t_{\mathrm{FT}}-i,$$

where $t_{\mathrm{FT}}$ is the time instant of FT (see Equation (2)), and *i* is expressed as

(24) $$\underset{i}{\mathrm{argmin}}\left|t_{\mathrm{FT}}-\left(j+{\mathrm{RUL}}_{j}\right)\right|\leq \varepsilon ,\;\forall j\in \left[i,t_{\mathrm{FT}}\right]$$

and ε is the admissible error bound.

## 4. Results

In this section, results based on the Barcelona case study, detailed in Section 2, are presented next to show the performance of the methodology proposed in this work.

The methodology presented has been tested off-line using real data from several past scenarios [27]. This work addresses the methodology that will be used on-line by the WU in a medium-term future, once the on-line requirements have been validated and analyzed.

The results presented here are focused on the prognosis module. The diagnosis module results are already presented in [17], showing anticipation of the sensor fault detection in about 12 days before the experts reported the sensor incidences. Thus, the data used by the prognosis module, to generate the results presented in this section, have been previously validated and processed by the diagnosis module.

The data used to generate the results come from the multi-parametric (chlorine, pH, temperature and conductivity) sensors (0794, 0795 and 0801), the chlorine analyzer X127701D and the incidences reported by the WU experts to the maintenance department (applied to the part of the Barcelona network presented in Figure 2).

The chlorine concentration observed is around 0.5 mg/L and the minimum value allowed by the Government of Catalonia regulation of chlorine concentration in the WDS is 0.2 mg/L. Hence, the minimum threshold to train the models is FT=0.2.

**Figure 4:**
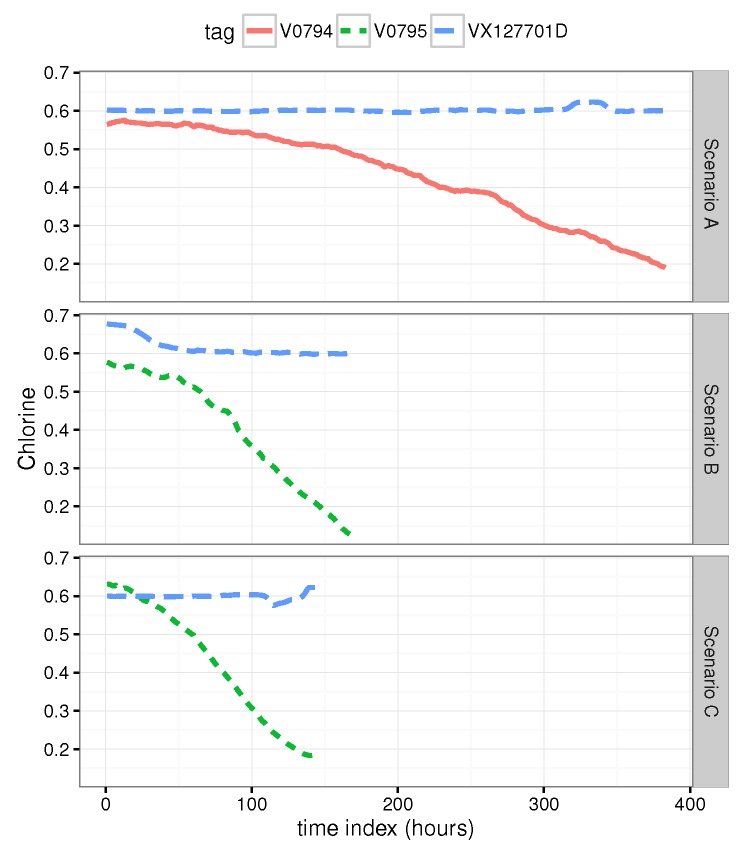
Fault scenarios of chlorine sensors.

The scenarios analyzed are three different chlorine decay scenarios. Figure 4 shows the three scenarios A, B and C, vertically stacked. The long-dashed blue line is the chlorine signal of VX127701D, the transport analyzer placed in the reservoir (see Figure 2). The dashed green line is the V0795 chlorine signal. The solid red line is the V0794 chlorine signal. As it can be noted, the chlorine decays are not equal in velocity and linearity. Scenario A shows a slow decay till 0.2 of chlorine in $t_{\mathrm{FT}}=378$ h (16 days) with some slumps. Scenario B shows a decay to 0.2 of chlorine in $t_{\mathrm{FT}}=147$ h (6 days). Scenario C shows a chlorine decay in $t_{\mathrm{FT}}=130$ h (5 days). Scenario B presents the most linear decay of them. While scenario C presents a slight curve at the end. As it will show next, these factors (slumps and non-linear decays) impacts directly on the prognosis performance.

**Figure 5:**
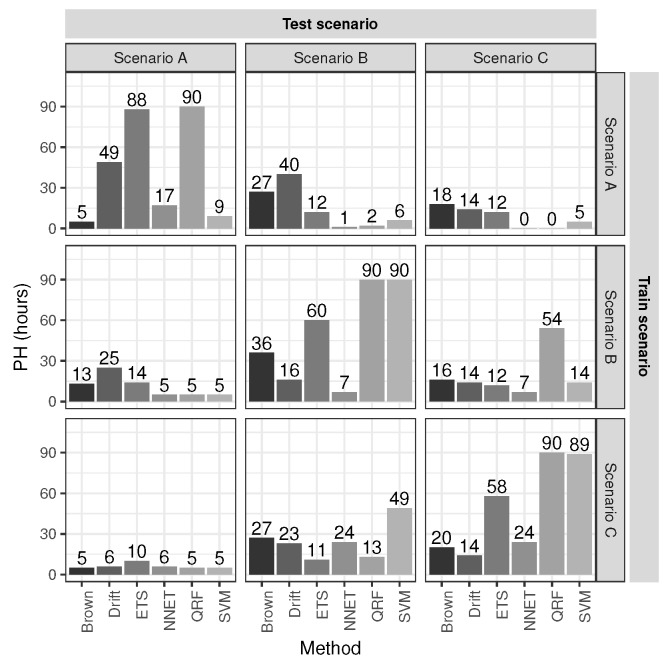
Evaluation of the prognosis performance using the PH metric.

The prognosis performance metric PH, Equation (23), have been evaluated on the six models detailed in Section 3 with ε=0.10×H and H=90, i.e., ε=9. As mentioned before, the models are trained using one scenario and evaluated with the others to avoid over-fitting and evaluate the generalization. Figure 5 shows the PH evaluation training each model with one scenario (stacked vertically) and tested with the others (stacked horizontally). The bar plots in the diagonal are the evaluation of the training data sets.

**Figure 6:**
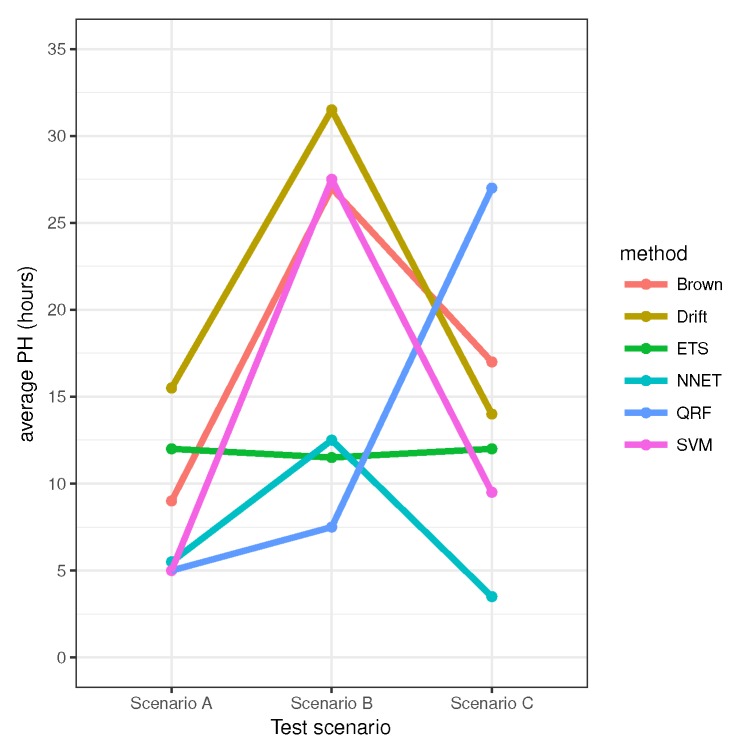
Average PH leaving out the scenarios that are the same for training and testing.

Finally, to summarize the performance results, Figure 6 shows the PH average for each testing scenario, and again leaving out the scenarios where training and testing are both the same in order to evaluate the generalization performance.

As can be noted, ETS, QRF and SVM algorithms show a good performance when the training and testing scenarios are both the same (see the diagonal results in Figure 5). However, the PH average in Figure 6, shows clearly the poor performance of ETS, NN and QRF methods when are applied to testing scenarios different than training scenarios, excluding QRF applied to scenario C. In contrast, drift and Brown methods have the best performance with highest PH averages in Figure 6. One relevant fact that can be observed in Figure 6 is the higher average performance obtained in scenario B by almost any model compared against in scenarios A and C. This is because the decay of scenario B is more linear than in A and C (see Figure 4) and therefore more predictable.

The bad performance of the models NN and QRF is due to the model construction process. These kinds of machine learning models require a lot of data, i.e., a large set of scenarios, to train them in order to generalize properly with new unseen scenarios. In this work, these models have been trained with only one scenario and tested with the others, therefore obtaining worst performance than Brown and drift models. With the exception of the SVM model, which uses only one scenario for training, and is able to perform similar to the Brown model.

The results of the first row of bar plots from Figure 5 are discussed below. Figures from 7 to 18 present the results obtained with the different results models trained with scenario A and applied to the scenarios B and C.

**Figure 7:**
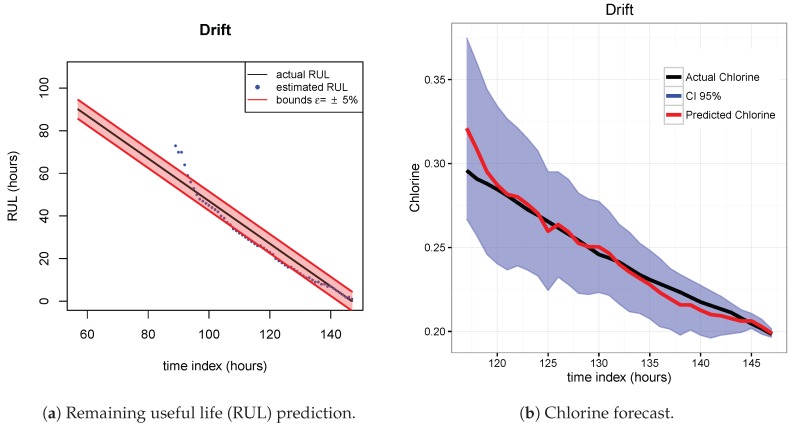
Drift model. Train scenario A and test scenario B.

**Figure 8:**
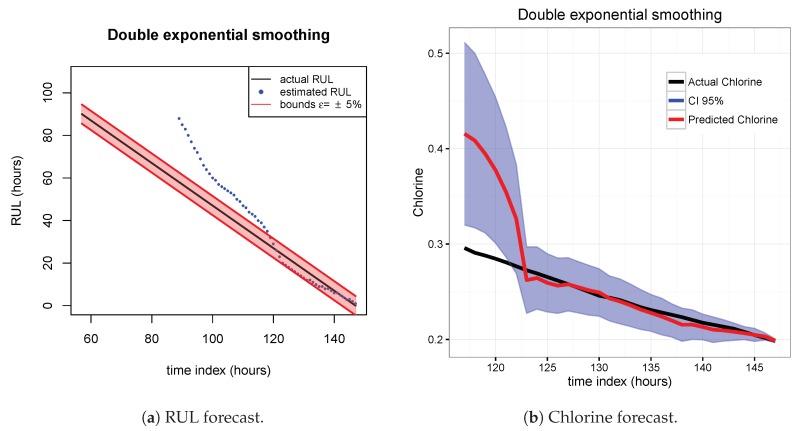
Brown model. Train scenario A and test scenario B.

**Figure 9:**
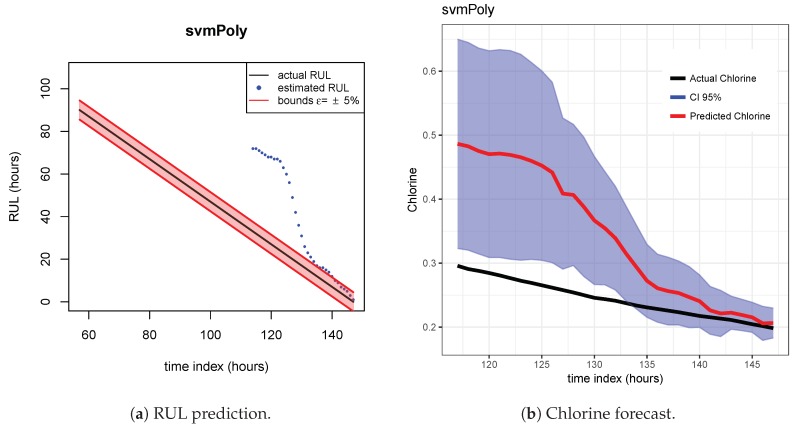
SVM model. Train scenario A and test scenario B.

**Figure 10:**
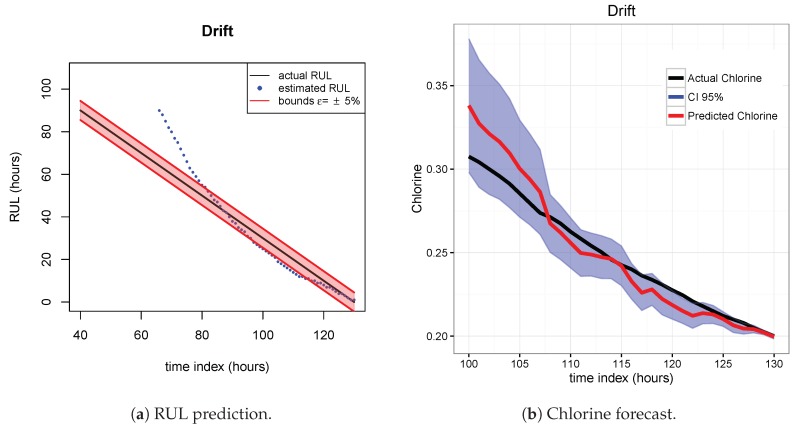
Drift model. Train scenario A and test scenario C.

**Figure 11:**
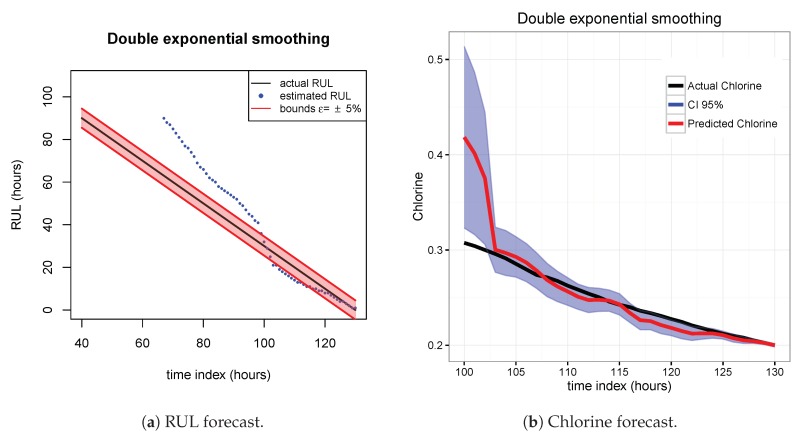
Brown model. Train scenario A and test scenario C.

**Figure 12:**
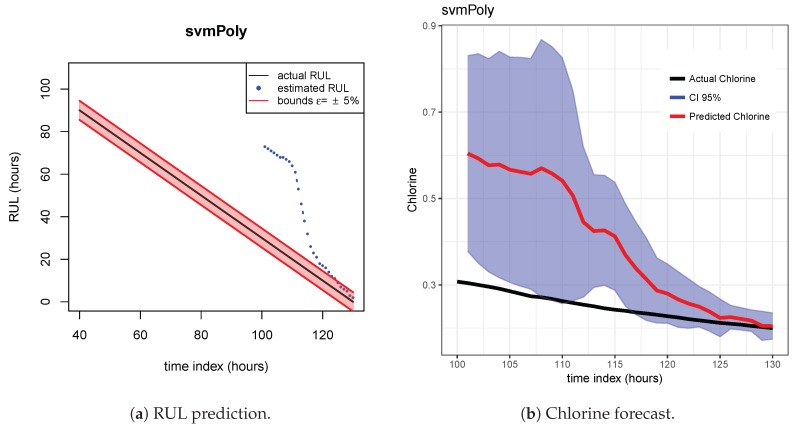
SVM model. Train scenario A and test scenario C.

Figure 7, Figure 8 and Figure 9 show the drift, Brown and SVM results when trained with scenario A and applied to scenario B. As commented before, this good performance is due to the linearity of the chlorine decay at the end of scenario B. In contrast, scenario A has small bumps at the end and scenario C has a slight curve leading to worse performances. Figure 10, Figure 11 and Figure 12 show the inferior performance on scenario C by the drift, Brown and SVM models, respectively.

**Figure 13:**
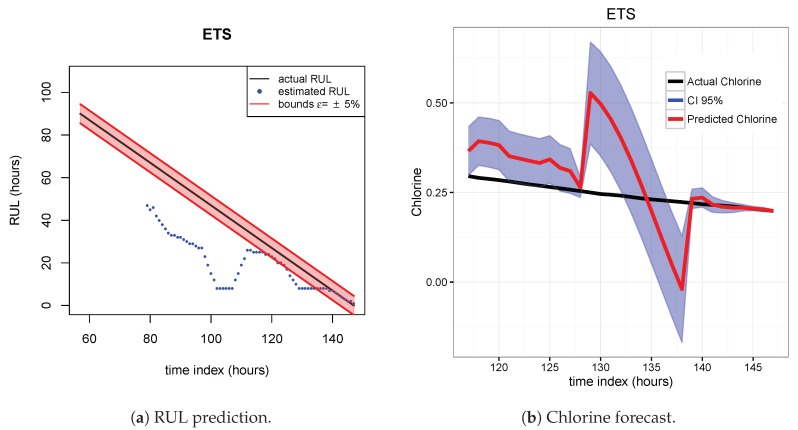
ETS model. Train scenario A and test scenario B.

**Figure 14:**
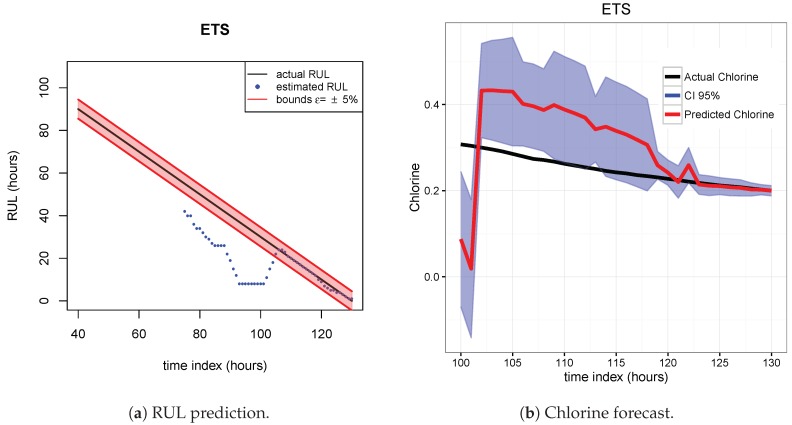
ETS model. Train scenario A and test scenario C.

**Figure 15:**
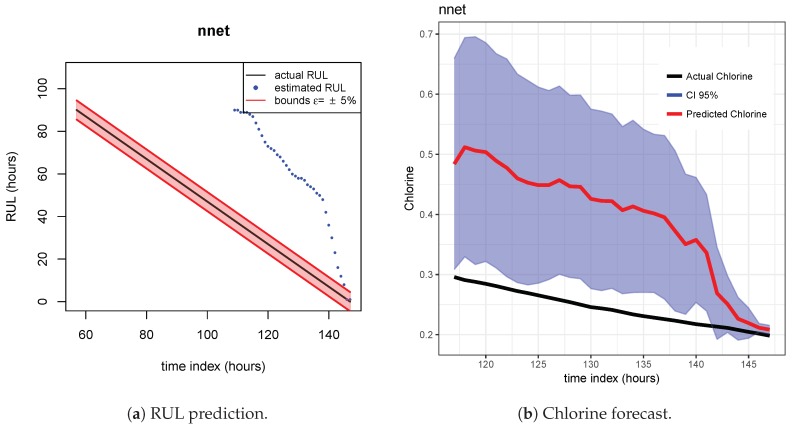
NN model. Train scenario A and test scenario B.

**Figure 16:**
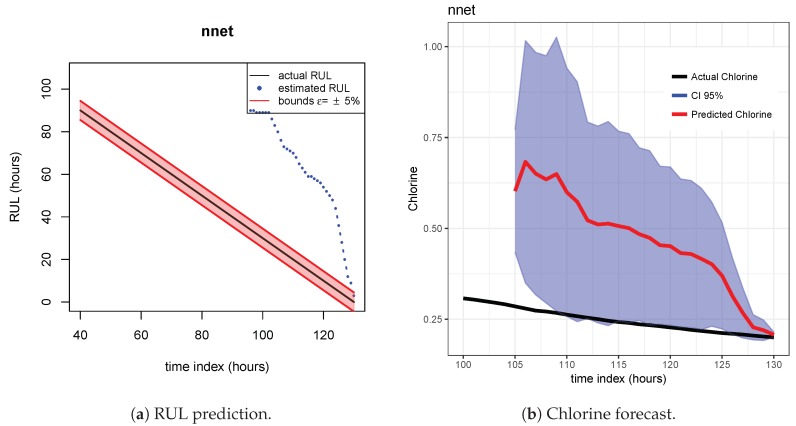
NN model. Train scenario A and test scenario C.

**Figure 17:**
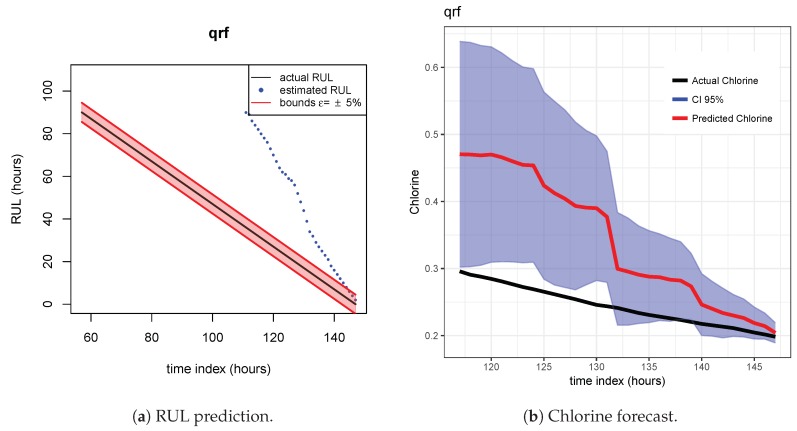
QRF model. Train scenario A and test scenario B.

**Figure 18:**
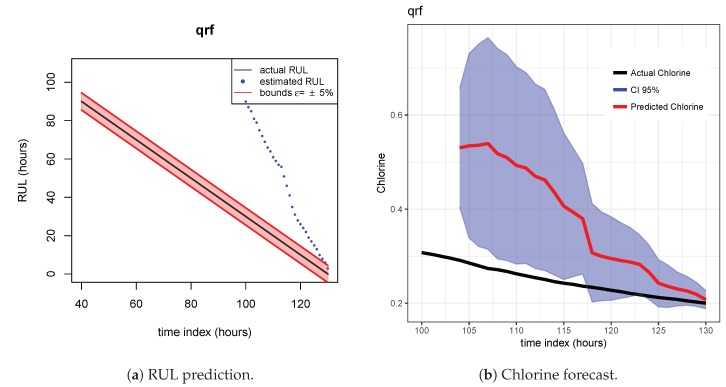
QRF model. Train scenario A and test scenario C.

As indicated before, ETS (Figure 13 and Figure 14), NN (Figure 15 and Figure 16) and QRF (Figure 17 and Figure 18) show a poor generalization.

## 5. Conclusions

This paper presents a prognosis approach for the water quality sensors using advanced data analytics approaches.

The complexity of chlorine sensors requires a regular maintenance plan to avoid monitor unreliable data and infer wrong conclusions. The prognosis framework presented can help the WU to predict these faulty states in order to apply predictive maintenance. Therefore, this allows decreasing corrective actions reducing OPEX costs of the WU.

On the one hand, a diagnosis framework has been briefly discussed that guarantees that no event or sensor fault is present before running the prognosis approach [17]. On the other hand, a prognosis framework has been presented to predict the RUL of chlorine sensors that presents a chlorine decay due to loss of sensitivity. The proposed prognosis approach has been assessed using three real scenarios from the Barcelona Water Network.

Brown and drift methods have shown a bad performance when non-linear shapes are present on the chlorine decay, such as bumps and curves. While the ETS method shows poor performance when applied to different scenarios that the trained one indicating an inherent over-fitting behavior. The drift method shows the best performance average, but Brown showing a slightly less performance average has less variance. For this reason, Brown is the one proposed to be used in the real implementation.

In contrast, the nonlinear models considered (NNET, QRF and SVM) do not provide the expected good results due to the reduced amount of data used for model construction. They require a larger number of training scenarios to generalize properly with new unseen scenarios.

The complexity of the model is an important requirement for the experts of the WU. Therefore, according to the performance and the simplicity of the implementation, the Brown method is the optimal choice for the prognosis module, discarding the other methods.

The methodology and the results detailed in this work have been presented to the experts of the WU. They expressed their approval and satisfaction with the results obtained. However, this work is a study phase of the methodology and it is not implemented on-line by the WU yet.

Finally, future work will deal with the on-line deployment of the proposed methodology. Moreover, many more decay scenarios in order to improve the machine learning model’s performance will be considered.

## Figures and Tables

**Table 1:** Problems affecting quality sensors.

Cause	Consequence
Communication problem	Data gap
Loss of sensitivity	Flat signal or slow drift down
Electronic malfunction	Noise and peaks
Miscalibration	Offsets

**Table 2:** Fault signatures of diagnosis indicators (residuals).

PD	ANN	$\bar{\mathrm{PD}}$ ∧ ANN	Cause
1	1	0	Sensor fault
1	0	0	Sensor fault
0	1	1	Quality event
0	0	0	Normal state
